# Chiral amplification in a cyanobiphenyl nematic liquid crystal doped with helicene-like derivatives

**DOI:** 10.3762/bjoc.5.50

**Published:** 2009-10-07

**Authors:** Alberta Ferrarini, Silvia Pieraccini, Stefano Masiero, Gian Piero Spada

**Affiliations:** 1 Dipartimento di Scienze Chimiche, Università di Padova, 35131 Padova, Italy; 2 Dipartimento di Chimica Organica “A. Mangini”, Alma Mater Studiorum – Università di Bologna, via San Giacomo 11, 40126 Bologna, Italy

**Keywords:** chirality, cholesteric, helical twisting power, helicene, nematic liquid crystal, Surface Chirality model

## Abstract

The addition of a chiral non-racemic dopant to a nematic liquid crystal (LC) has the effect of transferring the molecular chirality to the phase organization and a *chiral nematic* phase is formed. This molecular chirality amplification in the LC provides a unique possibility for investigating the relationship between molecular structure, intermolecular interactions, and mesoscale organization. It is known that axially chiral or helical-shaped molecules with reduced conformational disorder are good candidates for high *helical twisting power* derivatives. In particular, biaryl derivatives are known to be efficient chiral inducers in biaryl nematic mesophases. In this paper, we focus on a new series of helicene-like molecules of known absolute configuration. We have integrated cholesteric pitch measurements with geometry optimization by DFT calculations and analysis of the twisting ability by the Surface Chirality model to shed light on the structural features responsible for the analogies and differences exhibited by these derivatives. The investigation of these dopants with well-defined geometry, by virtue of the low conformational freedom, and the substituents variously distributed around the core, allows us to extend our knowledge of the molecular origin of the chirality amplification in liquid crystals and to confirm the simple relationship “molecular *P*-helicity” → “cholesteric *P*-handedness” for helical-shaped helicene-like derivatives.

## Introduction

**Figure 1:**
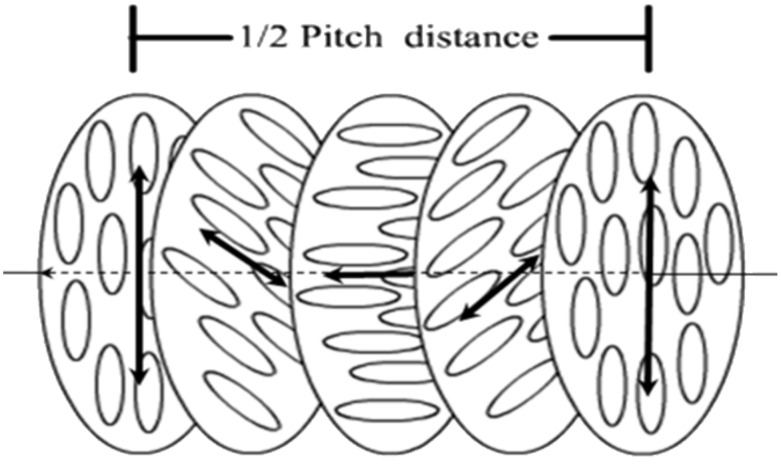
Schematic structure of a right-handed chiral nematic (cholesteric) phase. Black arrows represent the director, which rotates perpendicularly to an axis in a helical manner. Molecules (represented as ellipsoids) can take any orientation, but are preferentially aligned to the director.

*Nematic liquid crystals* (LCs) are fluid phases formed by anisometric molecules which, though free to rotate as in ordinary liquids, are preferentially aligned along a common axis, called *director*. The addition of a chiral non-racemic dopant to a nematic liquid crystal has the effect of transferring the molecular chirality to the phase organization and a *chiral nematic* (or *cholesteric*) phase is formed, in which the director rotates perpendicularly to an axis in a helical way (see Figure 1) [1].

For a given nematic host, the handedness and magnitude of the pitch of the cholesteric helix depend on the structure, concentration, and enantiomeric purity of the dopant. Enantiomeric pairs induce oppositely handed cholesteric phases. At low concentration, the helix pitch is inversely proportional to the molar fraction of the dopant; the propensity of a dopant to induce a helical organization in the LC matrix is then quantified by its *helical twisting power* (HTP), which is defined as [2–3]:

[1]
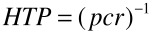


where *p* is the helical pitch of the cholesteric phase and *c* and *r* are the concentration (molar fraction) and the enantiomeric excess of the dopant, respectively. The sign of HTP is taken as positive if the induced cholesteric phase is right-handed (*P*).

Cholesteric induction has attracted great interest in the field of material science [4]. Most applications (for example, for the development of reversible optical memories) [5] require chiral dopants with good solubility in the nematic host and high helical twisting power. Understanding of the mechanism behind chirality transfer from solute molecules to host nematic phases is essential for designing LC-based chiral devices; our and other research groups have investigated this issue (for a selection of papers, see refs [6–21]). Cholesteric induction has also been exploited for the assignment of the absolute configuration of chiral molecules, as a viable alternative or complement to more usual techniques such as Circular Dichroism [2–3] (for a general review on CD, see refs [22–23]).

From a more fundamental point of view, the molecular chirality amplification in LC gives a unique possibility for investigating the relationship between molecular structure, intermolecular interactions, and mesoscale organization. The phenomenon of cholesteric induction can be explained in general terms as the result of the competition between (i) a chiral force, which originates from the chirality of intermolecular interactions and promotes a twist of the nematic director and (ii) an elastic restoring force, which can be traced back to the anisotropy of intermolecular interactions and opposes director distortions [24]. Different theories [25–27] have contributed to elucidate the molecular mechanism behind cholesteric induction, as well as Molecular Dynamics simulations [28–30]; for the connection between structure of the chiral dopant and cholesteric organisation, the *Surface Chirality* (SC) model has been shown to be particularly useful [31–32]. This is a phenomenological mean field theory wherein the anisotropy and chirality of the interactions of the dopant with the surrounding molecules are parameterized on the basis of the geometric features of the molecular surface. The underlying assumption is that short-range intermolecular interactions, which are modulated by molecular shape, are mainly responsible for the organization of thermotropic LCs. A valuable feature of the SC model is its realistic account of molecular structure; a detailed representation of the molecular surface can be easily obtained, once the atomic coordinates are known. The availability of reliable molecular structures is a requirement for the quality of HTP predictions. Nowadays, good estimates of molecular geometry can be obtained at an affordable computational cost using standard Quantum Mechanical (QM) tools.

It is known that axially chiral or helical-shaped molecules with reduced conformational disorder are good candidates for high HTP derivatives [2,4,6]. In particular, biaryl derivatives have been described as efficient chiral inducers in biaryl nematic mesophases (as, for instance, the widely used commercial mixture E7 from BDH) [33–45] and this has been viewed as a consequence of their structural analogy and molecular recognition via core–core interactions with the host molecules [46–47].

To avoid confusion with the *P*/*M* stereochemical descriptors of chirality axes, planes, or helices according to IUPAC nomenclature [48], we use here pseudo-*P* and pseudo-*M* to indicate the handedness of the twist between the two (aromatic) planes (for example, a biphenyl, irrespective of the presence of the substituents, is designed as pseudo-*P* when the two phenyl rings are arranged in such a way that a clockwise rotation (<90°) of the ring closer to the observer is required to obtain the coplanarity of the two aromatic planes). In most cases, (pseudo-*P*)-biaryls induce (*P*)-cholesterics; however, the relationship between stereochemical descriptor of the molecular chirality and handedness of the induced cholesteric phase is not straightforward. Indeed, it has been found that homochiral molecules with similar structures may induce cholesteric phases of opposite handedness [7,49]. A striking example is homochiral oligonaphthalenes, which, despite the clear structural helicity and the similar orientational behavior, exhibit no trivial relationship between molecular stereochemical descriptor (a*R* or a*S*) and cholesteric handedness (*P* or *M*) [50]. Changes in the molecular geometry, arising from the presence of substituents or conformational equilibria, can have a dramatic effect on the twisting ability of a dopant [2]. Moreover, in the case of dopants with low twisting power, cholesterics of opposite handedness may be induced in different LC solvents [8]. However, for solutes with clearly defined helicity and alignment axes, a weak sensitivity to small changes in structure and environment is more often observed. This uniformity of behavior was observed for the helicenes investigated; in penta- and hexa- and carbo- and hetero-helicenes, the relationship between the molecular stereochemical descriptor and the cholesteric handedness was verified and interpreted [51]: (*P*)-helicenes induce (*P*)-cholesterics in all the cases investigated.

**Figure 2:**
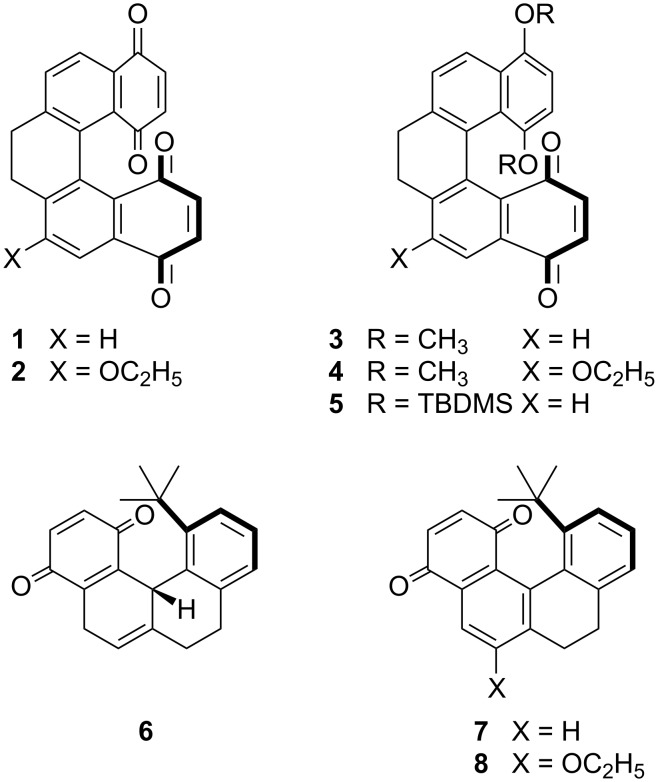
Structures of the dopants investigated.

In this paper, we focus on a new series of helicene-like molecules of known absolute configuration (Figure 2). We have integrated cholesteric pitch measurements with geometry optimization by DFT calculations and analysis of the twisting ability by the SC method to shed light on the structural features responsible for the analogies and differences exhibited by these derivatives. The investigation of these dopants with well-defined geometry, by virtue of the low conformational freedom, and substituents variously distributed around the molecular core, allows us to extend our knowledge of the molecular origin of the chirality amplification in liquid crystals.

## Results and Discussion

The enantioselective synthesis as well as the configuration assignment of enantiopure dihydro[5]helicene quinones or bisquinones **1–5** and of dihydro- (and tetrahydro-) [4]helicene quinones **6–8** has been described by Carreño, Urbano and coworkers [52–57].

The twisting powers of the helicenes under investigation measured in the nematic solvent E7 are reported in Table 1.

**Table 1:** Helical twisting power (HTP) measured in the nematic host E7 and chirality parameter *Q* calculated by the SC method.

Compound	HTP (µm^−1^)	*Q* (Å^3^)

**1**	+68	+14.8
**2**	+45	+20.6
**3**	+31	+8.3
**4**	+29	+9.3
**5**	+2.4	−0.1 (**I**) [0.44]^b^
		−4.4 (**II**) [0.37]
		+6.2 (**III**) [0.19]
**6**	+4.3^a^	−0.2
**7**	+9.2	+2.4
**8**	+5.6	+8.0

^a^HTP was measured for the enantiomer of **6**; therefore, the opposite of the measured value is reported in the table. ^b^Values refer to the three conformers of **5**, whose statistical weights are reported in brackets (see Figure 3).

We can see that all compounds possessing, as a common feature, homochiral *P*-helicity (for helical-shaped molecules, *P* and *M* describe the sense of the twist of the helix: this is right-handed, and is denoted as *P*, if the sense of the twist is clockwise as one progresses along the helix axis [48]) follow the relationship “molecular *P*-helicity” → “cholesteric *P*-handedness” already reported for a series of carbo- and hetero-helicenes [51]. (*P*)-7,8-Dihydro[5]helicene quinones or bisquinones **1–4** show moderate to high HTPs; despite the presence of the same tetracyclic core, derivative **5**, with the *tert*-butyldimethylsilyl (TBDMS) blocking group, exhibits the lowest measured HTP. This is a confirmation of the fact that bulky substituents, though lacking centres of chirality (or other stereogenic elements), can strongly affect the molecule-to-phase chirality transfer. The data in Table 1 also show that, unlike derivatives **1–4**, the [4]helicene quinones **6–8** have low twisting ability.

**Figure 3:**
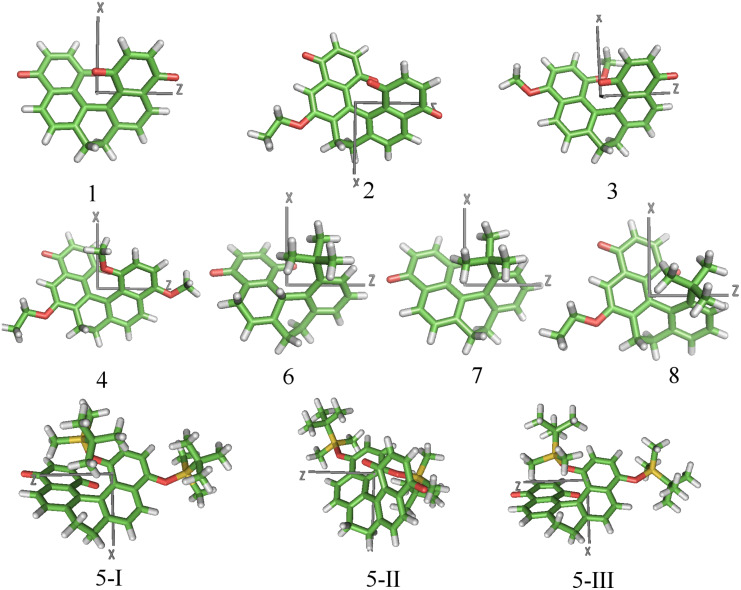
Geometry of dopants **1–8**. Structures were obtained from DFT calculations at the B3LYP/6-31g** level [58]. The *xz* plane is perpendicular to the molecular axis (*y*) with the lowest propensity to align to the local director, and *z* is the axis with the highest tendency to lie parallel to the director.

The structure of the dopants under investigation was determined by DFT calculations at the B3LYP/6-31g** level [58]; the optimized geometries are shown in Figure 3. In the dihydro[5]helicene quinones and bisquinones **1–5**, the naphthoquinone rings are approximately planar, with twist angles ranging between 42° and 43° for **1–4** and a slightly lower angle, around 40°, for **5**. A larger twist angle of 50.1° is found in dihydro[4]helicene quinones **7** and **8**, whereas a wider value, 81.7°, is allowed in tetrahydro[4]helicene quinone **6**, because of the higher flexibility arising from the presence of an additional dihydro ring. In all derivatives, the core of the fused rings is conformationally constrained, and for most of them, even the introduced substituents do not provide high conformational freedom. The O–C_Alkyl_ bond of alkoxy groups lies in the plane of the adjacent aromatic ring; for steric reasons, only the conformations shown in Figure 3 are allowed when a methoxy substituent is present. For **2**, **4**, and **8**, other structures, differing in the conformation of the ethoxy side chain, are also possible, but in view of their high energy, they have a negligible weight at room temperature. A different behavior is exhibited by derivative **5**, which has bulkier TBDMS substituents; one of them is constrained by the proximity of the quinone ring, whereas the other is pointing towards the molecular periphery and is more free. Three structures with similar energy were obtained, differing essentially in the value of the torsional angle for the O–Si bond of the less hindered *tert*-butyl-silyl group. These structures are also displayed in Figure 3.

The twisting ability of derivatives **1–8** was analysed according to the SC method [31–32]; molecular surfaces generated on the basis of the optimized geometries [59] were used. Within this approach, the HTP of a chiral dopant in a nematic solvent is proportional to the so-called *chirality parameter Q*, which holds the coupling of the chirality and orientational order and is proper of each dopant; the *chirality parameter Q* is defined as:

[2]
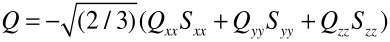


where *S_ii_* is the *i*th cartesian component of the Saupe ordering matrix, which specifies the degree of alignment to the local director of the *i*th molecular axis, and *Q_ii_* quantifies the helicity of the molecular surface, as viewed along the same axis. The proportionality factor *A* between HTP and *Q* depends on the macroscopic properties of the host, being defined as *A* = *RT* ξ/2π *K*_22_ ν_m_, where *T* is the absolute temperature, *R* is the gas constant, *K*_22_ and ν_m_ are the twist elastic constant and the molar volume of the liquid crystal host, respectively, and the parameter ξ is the orienting strength of the medium (and is related to the degree of order of the host).

**Figure 4:**
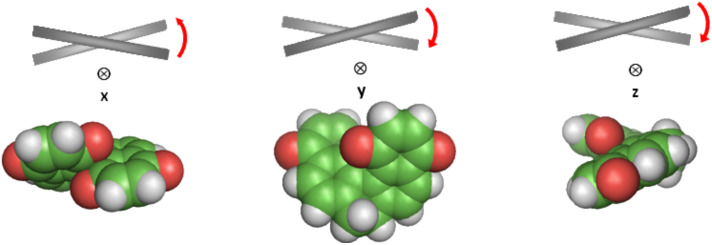
Helicity of the molecular surface of derivative **1** along its principal alignment axes in the liquid crystal environment.

The principal elements of the ordering matrix **S** calculated for derivatives **1–8** are reported in Table 2. The *x*, *y*, *z* labels denote the principal alignment axes in the molecule; in particular, *z* and *y* identify the molecular directions with the strongest tendency to align parallel and perpendicular to the local director, respectively. These directions, which are univocally identified once the ordering matrix **S** is calculated, are shown by the axes superimposed on the chemical structures in Figure 3. We can see that in most of the cases *y* lies close to what can be considered as the ‘molecular helix axis’, whereas the *xz* plane corresponds to what can be approximately defined as the ‘molecular plane’. The *z* axis, which is preferentially aligned to the director, lies in the direction of maximum molecular elongation, which for most derivatives is close to that of the aryl–aryl bond; for dopants with twofold rotational symmetry, the *x* direction is parallel to the *C*_2_ axis. To understand the orientational behavior of dopants, we must recall the relationship −0.5 ≤ *S_ii_* ≤ 1, with *S_ii_* = 1 and *S_ii_* = −0.5 denoting perfect alignment of the *i*th molecular axis parallel and perpendicular to the director, respectively [31]. Thus, the *S_ii_* values reported in Table 2 indicate that all the derivatives have a similar orientational behavior in the nematic host. The relatively low *S_zz_* values say that these dopants are not strongly ordered in the liquid crystal host; more important for their twisting ability, they tend to keep the ‘molecular helix axis’ perpendicular to the director, whereas the latter preferentially lies on the ‘molecular plane’. As a consequence, our dopants are expected to induce a cholesteric phase with the same helicity as that characterising the ‘molecular helix axis’ (*y* in Figure 4).

**Table 2:** Principal elements of the Saupe ordering matrix, **S**, and corresponding elements of the chirality tensor **Q**. Axis labels are shown in Figure 3.

Compound	*S_xx_*	*S_yy_*	*S_zz_*	*Q_xx_* (Å^3^)	*Q_yy_* (Å^3^)	*Q_zz_* (Å^3^)

**1**	0.02	−0.29	0.27	−89.5	72.4	17.1
**2**	0	−0.32	0.32	−89.2	84.7	4.5
**3**	−0.05	−0.29	0.34	−83.3	67.1	16.2
**4**	−0.07	−0.31	0.37	−117.3	92.0	25.3
**5-I**^a^	−0.15	−0.19	0.34	−7.0	6.3	0.7
**5-II**	−0.16	−0.20	0.36	−62.5	48.1	14.4
**5-III**	−0.06	−0.26	0.32	−28.8	32.0	−3.2
**6**	−0.03	−0.18	0.21	−76.5	47.7	28.8
**7**	0.02	−0.22	0.20	−68.0	37.0	31.0
**8**	0.03	−0.26	0.23	−48.0	39.8	8.2

^a^Values refer to the three conformers of **5**, with different conformations around the O–Si bond of the less hindered TBDMS group (see text and Figure 3).

The *Q_ii_* values reported in Table 2 quantify the magnitude and sign of the helicity of the molecular surface, as seen along the *x*, *y*, *z* axes. Since the relationship *Q_xx_* + *Q_yy_* + *Q_zz_* = 0 holds [31], any molecule is characterized by the helicities of opposite signs, depending on the direction along which the molecular surface is viewed. This is not in contradiction with the presence of a well-defined stereochemical descriptor, because a given axis is chosen for such a definition by convention. We can see in Table 2 that for all dopants with the exception of one conformer of **5**, the molecular surface has negative helicity along the *x* axis, whereas the helicity is positive along the *y* and *z* axes. This is in agreement with the *P* helical stereochemical descriptor of the chiral dopants under investigation, which is defined with respect to the helix axis (*y*).

In the case of derivative **5**, order and chirality properties are not dictated by the central core, but seem to be significantly affected by the bulky substituents. Table 2 shows that different order parameters are predicted for the **I–III** conformers of **5**, which are also different from those of the other dopants under investigation. We can see even more substantial differences between *Q_ii_* components, with particularly low values predicted for the **5-I** conformer.

As a result of the surface chirality and the orientational behavior, induction of right-handed cholesterics is predicted for our dopants. The trend of the chirality parameter *Q* reported in Table 1 mirrors that of the measured HTPs, with more discrepancy for the weaker cholesteric inducers. A negative *Q* is obtained for **6** and the *Q* value calculated for **5** as a weighted average over all conformers is also negative. However, these negative values are very small and we can see that they are obtained just for the two derivatives with the lowest measured HTPs. Indeed, the twisting ability of a given dopant results from a delicate balance of chirality and anisotropy of dopant–host interactions and predictions of small effects would require a very detailed modelling of all intermolecular interactions. The SC method is particularly suitable for dopants with relatively high HTP, whose behavior is dominated by short-range interactions with the host. In general, low *Q* values may not be in agreement with measured HTPs. Another possible reason for this discrepancy, besides the neglect of long-range intermolecular interactions, such as the electrostatic ones, might be the molecular geometry used for *Q* calculation: in the absence of a net prevailing term, even relatively small geometry changes can modify the balance between the positive and negative contributions.

According to our SC model results, the low HTP measured for **5** and **6** has a different explanation: in the latter case, it simply reflects the low twisting ability of the dopant, whereas in the former, it comes from the cancelling effect of conformers which individually would induce a left-handed (**5-II**) and a right-handed twist (**5-III**). Moreover, inspection of Table 2 suggests that the lower twisting ability, measured and predicted for all the [4]helicene quinones **6–8** can be ascribed to weaker orientational order and lower helicity along the *y* axis. Both effects can ultimately be traced back to the larger dimensions of the [4]helicene quinone derivatives, which possess a wider extension of aromatic rings, capable of establishing stronger dispersion interactions with the host molecules. As to the effect of substituents, we can compare the results obtained for the pairs **1–2**, **3–4**, and **7–8**, which differ by the replacement of a phenyl hydrogen by an ethoxy group. The measured HTPs are smaller for the derivatives with the ethoxy substituent, whereas the opposite change is predicted from the SC calculations; the discrepancy between calculations and experiments is especially evident in the case of derivative **8**. These differences might indicate a role of electrostatic solute–solvent interactions, which are neglected in our model. It was already observed that these can be considered as a generally small, but non-negligible, correction to the underlying short-range interactions; their relative contribution can become relevant in the case of dopants with otherwise small twisting ability [7].

## Conclusion

Chirality is a peculiar molecular feature and its manifestations elude any trivial interpretation: different, often completely uncorrelated, responses depending on the experiment used are obtained in the attempt to quantify it.

The helical twisting power cannot simply be correlated with a global stereochemical descriptor of the molecule, as demonstrated for homochiral series of propeller-like heptalenes and oligonaphthalenes for which the handedness of the induced cholesteric depends critically on the substituents attached to the chiral core [49–50].

The investigation presented here confirms that, as already found for helicene derivatives [51], the simple relationship “molecular *P*-helicity” → “cholesteric *P*-handedness” exists for helicene-like compounds, in the absence of bulky and highly flexible substituents. Not surprisingly, taking into account molecular shape, the orientational behavior of **1–8** derivatives is analogous to that of bridged binaphthyls and also the helical twisting power can be interpreted in a similar way: the outcome is that pseudo-*P* dopants induce a right-handed (*P*)-cholesteric phase. The results obtained for the dopants investigated in the present work, with a clear molecular *P*-helicity and low conformational freedom, differing from each other in the presence of variously distributed substituents, confirm that short-range intermolecular interactions, parameterizable according to the molecular surface, are the main determinants of cholesteric induction in thermotropic liquid crystals. Other interactions, ascribable to the presence of electronegative groups, though present, are less relevant, and can have non-negligible effects in the case of dopants with low twisting ability.

## Experimental

### Helical twisting power measurements

Pitches and handedness of the cholesteric solutions in E7 have been obtained at room temperature using the lens version of the Grandjean-Cano method [60]. E7 from BDH is a commercial mixture of 4′-pentyl-, 4′-heptyl-, 4′-octyloxy-, and 4′-(4-pentylphenyl)-4-biphenylcarbonitrile in a 51:25:16:8 wt ratio (*T*_i_ 60 °C). The standard error of the pitch determination is ca. 10%. The technique is described in detail in ref [61].

### Geometry optimization

Calculation of the chirality parameter *Q* requires the molecular surface, which is generated on the basis of atomic coordinates. Since the chirality of the molecular surface strongly depends on the molecular geometry, accurate structures are needed to obtain reliable estimates of the twisting ability. The geometry of the molecules listed in Table 2 was obtained by quantum mechanical optimization with DFT at the B3LYP/6-31g** level [59].

### Chirality parameter and Saupe matrix calculation

The Saupe ordering matrix **S** and the chirality parameter *Q* of dopants were calculated as explained in refs [31–32]. Once the atomic coordinates were obtained, the molecular surface (the surface generated by rolling a spherical probe on the assembly of van der Waals spheres centred at the nuclear positions and approximated by a set of triangles, obtained with the algorithm developed by Sanner et al. [59]) was computed. The results reported in this work were obtained by setting the orienting strength ξ to 0.025 Å^−2^ and the rolling sphere radius to 3 Å [32]. The van der Waals radii *r*_H_ = 1 Å, *r*_C_ = 1.85 Å, *r*_O_ = 1.5 Å, and *r*_Si_ = 2.1 Å were used [62]. A density of points equal to 5 Å^−2^ was assumed for the molecular surface.
